# MIS-TLIF revision surgery for postoperative interbody fusion cage migration via contralateral intervertebral foramen approach: A case report

**DOI:** 10.1097/MD.0000000000047252

**Published:** 2026-01-23

**Authors:** Qipeng Xia, Qi Chen, Jingxiang Chen, Hongtao Xu, Tianlong Wu, Jiangjun Zhou, Jianping Wang, Min Zhao

**Affiliations:** a Department of Orthopedics, Yingtan People’s Hospital, Yingtan, Jiangxi, China; b Department of Orthopedics, Second Affiliated Hospital, Jiangxi Medical College, Nanchang University, Nanchang, Jiangxi, China; c Department of Orthopedics, Affiliated Hospital of Jiangxi University of Traditional Chinese Medicine, Nanchang, Jiangxi, China; d Department of Orthopedics, The 908(th) Hospital of Chinese People’s Liberation Army Joint Logistic Support Force, Nanchang, Jiangxi, China.

**Keywords:** contralateral intervertebral foramen access, fusion cage migration, MIS-TLIF, revision surgery

## Abstract

**Rationale:**

Minimally invasive transforaminal lumbar interbody fusion (MIS-TLIF) is widely applied for lumbar spine disorders due to reduced surgical trauma and faster recovery. However, postoperative interbody fusion cage migration remains a challenging complication. This report introduces a contralateral intervertebral foramen revision strategy as an alternative solution to correct cage migration without removing the original cage.

**Patient concerns:**

A 55-year-old Asian female presented with progressive lower back pain, radiating leg pain, and limited mobility 5 weeks after MIS-TLIF. Symptoms significantly affected daily activities and were consistent with neurological compression.

**Diagnoses:**

Postoperative cage migration with associated nerve compression was confirmed by physical examination and imaging studies, including X-ray, computed tomography, and magnetic resonance imaging.

**Interventions:**

A revision MIS-TLIF procedure was performed via the contralateral intervertebral foramen. The migrated cage was repositioned using the original cage handle and Kirschner wire, and an additional interbody cage was implanted to enhance segmental stability. This approach avoided removing the original cage and minimized anatomical disruption.

**Outcomes:**

Neurological symptoms improved immediately after surgery, and postoperative imaging confirmed satisfactory cage correction and stabilization. At the 1-year follow-up, the patient reported no recurrent discomfort, and computed tomography imaging demonstrated stable cage position and interbody fusion.

**Lessons:**

Contralateral intervertebral foramen access in MIS-TLIF revision surgery offers a minimally invasive alternative for cage migration management, avoiding the need for cage removal and reducing operative trauma. This technique may provide a viable option for similar revision scenarios requiring neural decompression and cage repositioning.

## 1. Introduction

Transforaminal lumbar interbody fusion (TLIF) is a widely used technique for treating degenerative lumbar diseases and lumbar spondylolisthesis, offering effective decompression and fusion through a unilateral approach. Minimally invasive transforaminal lumbar interbody fusion (MIS-TLIF), utilizing a fixed surgical channel, further reduces paraspinal muscle trauma, blood loss, recovery time, and infection risk, leading to its broad adoption.^[1,2]^ Fusion cage displacement is a significant complication following TLIF or MIS-TLIF, with potential for posterior, anterior, or lateral migration.^[3]^ Posterior migration into the spinal canal or intervertebral foramen is particularly concerning due to the risk of neurological damage and compromised fusion. Reported incidence rates of fusion cage displacement post MIS-TLIF range from 0.35% to 6%.^[4,5]^ If displacement results in nerve compression or instability, revision surgery may be necessary, though it is technically challenging due to scar tissue formation around the spinal cord and nerves, increasing the risk of neurological injury.^[6]^Common approaches include anterior, posterior, lateral, transforaminal, and endoscopic-assisted methods.^[7–9]^ This report presents a case of posterior cage migration after MIS-TLIF, successfully treated with an innovative contralateral intervertebral foramen approach, leading to satisfactory cage repositioning and patient outcomes.

## 2. Case report

**Figure 1.**
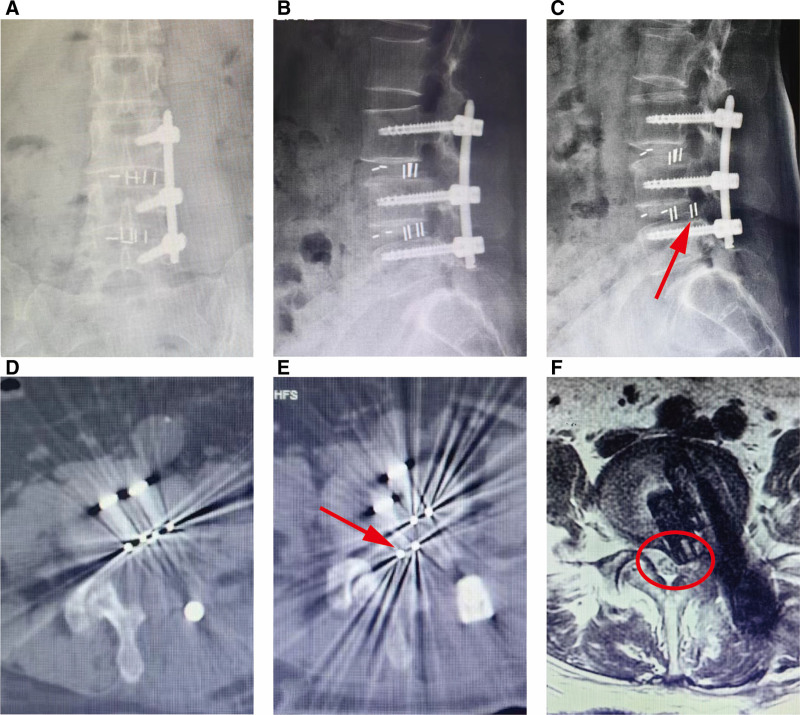
X-ray images at postoperative day 5 (A, B) and 5 weeks (C) after the initial surgery. Cross sectional CT images of the L4-5 segment at postoperative day 5 (D) and 5 weeks (E) following the initial surgery. (F) Transverse MRI scan images of the L4-5 intervertebral space. CT = computed tomography, MRI = magnetic resonance imaging.

A 55-year-old Asian female undergone MIS-TLIF at the L3-4 and L4-5 levels due to L3 vertebral spondylolisthesis and L4-5 disc herniation, which relieved her left lumbosacral pain. Postoperative imaging confirmed correct placement of the interbody cage (Fig. 1A, B, and D). However, 5 weeks after surgery, she developed worsening low back pain accompanied by pain in the left thigh and numbness in the left calf, which were aggravated by walking or bending to the left. Physical exams revealed a positive straight leg raise test, reduced sensation in the left L5 dermatome, and weakened left extensor hallucis longus. Blood tests were normal, ruling out infection. Follow-up imaging demonstrated posterior and leftward migration of an interbody cage into the spinal canal, compressing the nerve roots (Fig. 1C, E, and F). After thorough discussion, the surgical team decided to perform a revision MIS-TLIF via the contralateral approach, aiming to reposition the migrated cage while minimizing the trauma and risk of neural injury associated with reoperation.

**Figure 2.**
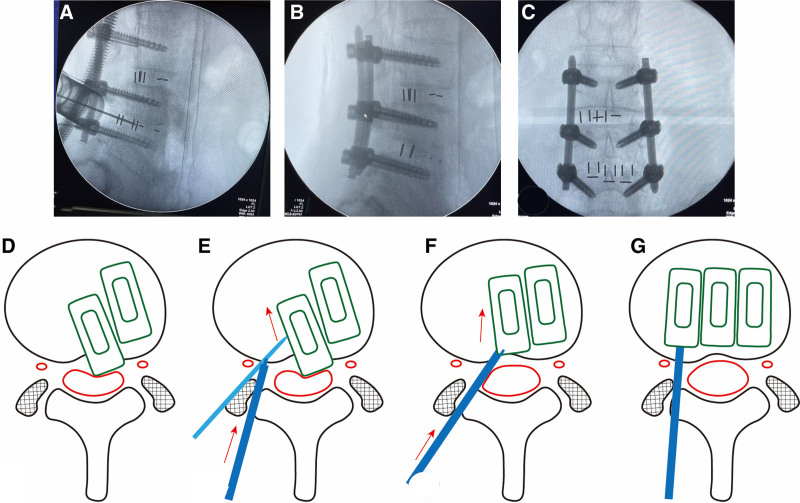
(A) After the insertion of the Kirschner wire, X-ray imaging was used for positioning. (B, C) After the surgery, X-ray images were taken from both the frontal and lateral perspectives for evaluation. (D-G) Surgical procedure schematic. Expose the intervertebral foramen by resecting the small joint and partial lamina (D). Adjust and reposition the fusion cage using the Kirschner wire (light blue) and the fusion cage holder (dark blue) (E, F). Insert the 3rd fusion cage (G).

The surgical procedure was carried out following these steps:

1. Following general anesthesia, the patient was positioned prone on a fluoroscopy surgical table. Using a C-arm X-ray machine, the L4-5 segment was localized, and the pedicle was marked. Routine disinfection and draping were performed.
2. A guide wire for pedicle screw insertion was percutaneously inserted. A longitudinal incision of about 3 cm was made at the marked location based on the Wiltse approach. The skin, subcutaneous tissue, and deep fascia were sequentially incised, followed by direct separation of the multifidus and longest muscles to reach the articular process. Graduated dilators were then inserted, and a fixation channel device (Spine, Double Medical, China) was installed.^[10]^
3. Confirmation of the correct surgical segment was done through fluoroscopy. The upper and lower facet joints were exposed, along with the upper and lower vertebral plates. Partial removal of the L4 vertebral body’s lower plate was performed until the upper edge of the ligamentum flavum was exposed. Subsequently, the ligamentum flavum was dissected, and the upper edge of the L5 vertebral body’s upper plate was completely removed. After adequate exposure of the intervertebral foramen, the dura mater and nerve roots were protected using 2 pieces of cotton and a nerve hook to expose the displaced fusion cage (Fig. 2D).
4. A fine Kirschner wire was inserted into the small hole on the side of the fusion cage (Fig. 2A and E). The fusion cage holder was attached to the Kirschner wire and gently tapped (Fig. 2E). As tapping progressed, the position and angle of the fusion cage changed, and fluoroscopy revealed the marker of the fusion cage moving forward into the intervertebral space. Tapping continued to adjust the angle of the displaced fusion cage until the posterior edge of the fusion cage could be exposed. The Kirschner wire was then removed, and the fusion cage holder was attached to the posterior edge corner slot of the fusion cage (Fig. 2F). Continued tapping was performed to drive the displaced fusion cage into the appropriate position. To prevent the inner fusion cage from slipping backward again, a 3rd fusion cage was inserted and adjusted to snugly fit against the inner fusion cage (Fig. 2G).
5. Pedicle screws were inserted along the guide wire at the L3-5 levels, followed by another C-arm machine fluoroscopy to ensure proper internal fixation and positioning of the fusion cage (Fig. 2B and C). Finally, cosmetic skin closure of the incision was performed using absorbable sutures.

**Figure 3.**
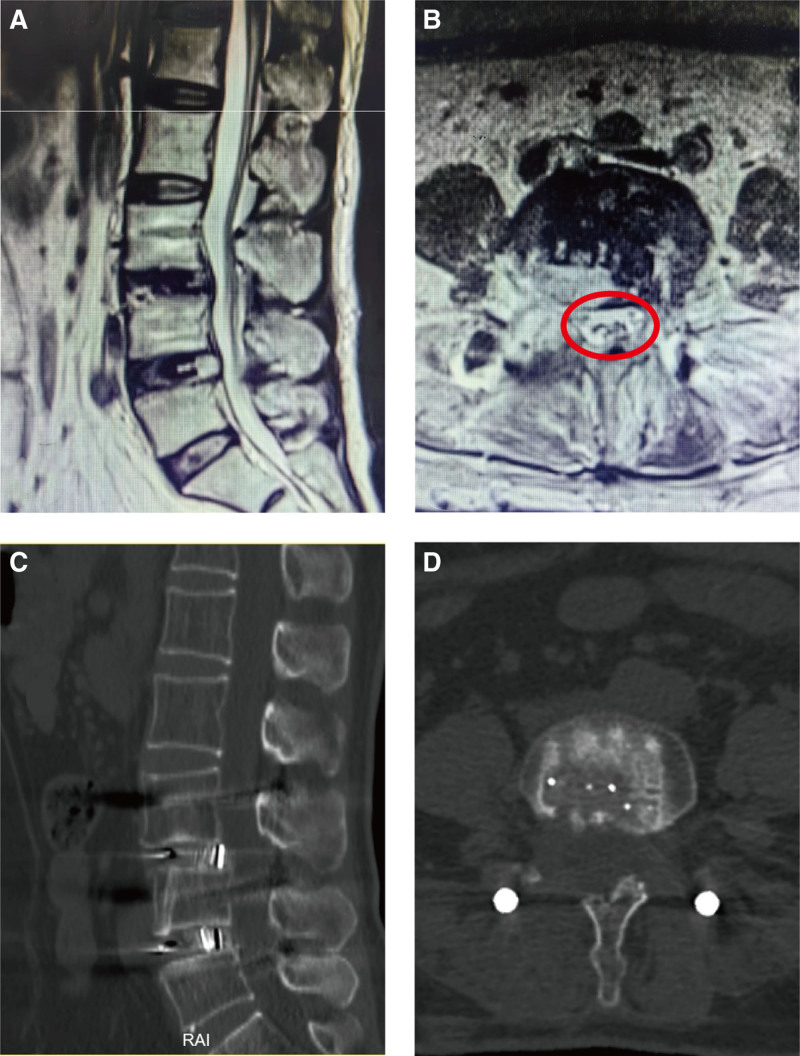
MRI of the lumbar spine for postoperative review at 3 days post-surgery (A, B). CT scan of the lumbar spine for the 1-year postoperative follow-up (C, D). CT = computed tomography, MRI = magnetic resonance imaging.

The patient experienced significant symptomatic improvement after surgery. Magnetic resonance imaging demonstrated complete resolution of spinal nerve compression (Fig. 3A and B). At the 1-year postoperative follow-up, the patient remained asymptomatic except for mild lower back weakness. Computed tomography scans confirmed appropriate positioning of the fusion device without significant displacement, and solid fusion was observed within the intervertebral space (Fig. 3C and D).

## 3. Discussion

MIS-TLIF has gained popularity due to its advantages of reduced tissue trauma, lower surgical risks, shorter hospital stays, and faster postoperative recovery. As with other fusion techniques, accurate placement of the interbody cage is critical for achieving successful intervertebral fusion. Cage migration is a notable complication, most commonly occurring within the first 3 months after surgery. Identified risk factors include vertebral endplate damage, screw loosening, and postoperative infection.^[11]^

In the present case, we speculate that the potential causes of cage migration included: insufficient depth of cage insertion or a posteriorly positioned interbody cage during implantation; inadequate stability due to unilateral fixation in the initial surgery; and excessive postoperative activity by the patient, such as frequent stair climbing. Blood tests and imaging studies at the time of admission ruled out infection.

Most patients with fusion cage displacement require open surgical repair.^[12]^ However, due to scar tissue in the surgical area and prior internal fixation, revision surgery has always been a technical challenge, with limited reports available. Some scholars suggest that anterior lumbar interbody fusion is a viable revision technique for addressing fusion cage displacement following TLIF or posterior lumbar interbody fusion procedures, as it effectively removes the previous fusion cage and offers optimal sagittal plane correction.^[13]^ Other effective methods include oblique lumbar interbody fusion and endoscopic techniques, although their suitability depends on the individual patient’s circumstances. For example, Xu et al described an endoscopic revision technique for fusion cage posterior retreatment after TLIF, using a high-speed burr to remove the protruding posterior edge of the cage within the spinal canal, effectively relieving neurological compression.^[6]^ Additionally, endoscopic techniques have proven effective in addressing the lateral displacement of fusion cages post-oblique lumbar interbody fusion surgery.^[14]^

In this case, the retreated fusion cage is centrally positioned, while the unaffected fusion cage on the left outer side may obstruct the surgical field during revision surgery. Additionally, scar tissue adhesion within the spinal canal may complicate the procedure. Therefore, performing revision surgery via the original surgical approach, midline approach, or left lateral approach would significantly increase the difficulty, trauma, and risk of nerve injury. Endoscopic techniques, while effective in grinding down the protruding portion of the displaced fusion cage, could generate vibrations that might loosen the unaffected fusion cage on the outer side, thereby increasing the risk of nerve injury. Considering these challenges, we explored the feasibility of correcting the retreated fusion cage without removing it, thereby relieving nerve compression. By understanding the structural features of the fusion cage material, we identified multiple small holes on its lateral side that could serve as points of leverage for surgical instruments. Computed tomography scans revealed that the posterior edge of the retreated fusion cage is parallel to the intervertebral foramen, with a relatively short distance from the contralateral intervertebral foramen to the lateral holes of the retreated fusion cage. This suggests that an approach from the contralateral intervertebral foramen could correct the retreated fusion cage without manipulating the unaffected fusion cage on the left side or replacing it with a larger cage. Additionally, due to inadequate stability on one side, the revision surgery required additional reinforcement fixation on the opposite side, addressing both issues simultaneously through a unified approach. This approach was validated through ex vivo simulation surgery.

All steps proceeded smoothly during the surgical procedure according to the preoperative plan. After successfully repositioning the migrated cage, we implanted a 3rd interbody cage adjacent to the relocated one to further enhance segmental stability. This strategy increased the overall contact area between the cages and the vertebral endplates, thereby facilitating intervertebral fusion.

During the contralateral foramen approach, particular attention was devoted to preserving the paraspinal muscles. Although a certain degree of muscle exposure was unavoidable, we minimized detachment and employed a muscle-splitting trajectory to reduce iatrogenic injury. Protecting the multifidus and surrounding paraspinal musculature is critical for maintaining postoperative spinal stability and promoting functional recovery, as muscle impairment or fatty infiltration has been associated with complications such as instrumentation loosening and suboptimal clinical outcomes.^[15]^ Despite the required contralateral exposure, this minimally invasive approach still offers significant advantages over traditional open revision surgery, including reduced surgical trauma, shorter hospitalization, and lower medical costs. The patient recovered well and expressed high satisfaction with the overall treatment process.

## 4. Conclusion

In summary, for the case presented, where symptomatic fusion cage displacement occurred following MIS-TLIF surgery, revision surgery performed via contralateral intervertebral foramen access successfully corrected the displaced fusion cage, relieving spinal nerve compression without the need for removal. This technique offers several advantages, including minimal invasiveness, rapid recovery, and high safety. It not only proves to be an effective method for correcting fusion cage displacement after TLIF surgery but also shows promise for addressing similar scenarios involving neural decompression.

## Acknowledgments

We sincerely thank all the medical staff involved in the diagnosis and treatment of this case.

## Author contributions

**Data curation:** Qipeng Xia, Jingxiang Chen, Hongtao Xu.

**Writing – original draft:** Qipeng Xia, Qi Chen.

**Writing – review & editing:** Qipeng Xia, Qi Chen, Min Zhao.

**Methodology:** Qi Chen, Tianlong Wu, Jiangjun Zhou, Jianping Wang.

**Conceptualization:** Min Zhao.

**Project administration:** Min Zhao.

## References

[1] Bakhsheshian J Khanna R Choy W. Incidence of graft extrusion following minimally invasive transforaminal lumbar interbody fusion. J Clin Neurosci. 2016;24:88–93. 26578209 10.1016/j.jocn.2015.09.005

[2] Phan K Rao PJ Kam AC Mobbs RJ. Minimally invasive versus open transforaminal lumbar interbody fusion for treatment of degenerative lumbar disease: systematic review and meta-analysis. Eur Spine J. 2015;24:1017–30. 25813010 10.1007/s00586-015-3903-4

[3] Han Z Ren B Zhang L. Finite element analysis of a novel fusion strategy in minimally invasive transforaminal lumbar interbody fusion. Biomed Res Int. 2022;2022:4266564. 35601152 10.1155/2022/4266564 PMC9117058

[4] Liu K Chang H Wang L Wang C Chen T Meng X. Risk factors for cage retropulsion after lumbar interbody fusion: systematic review and meta-analysis. World Neurosurg. 2019;132:273–81. 31521758 10.1016/j.wneu.2019.09.019

[5] Zhou Z-J Xia P Zhao F-D Fang X-Q Fan S-W Zhang J-F. Endplate injury as a risk factor for cage retropulsion following transforaminal lumbar interbody fusion: an analysis of 1052 cases. Medicine (Baltim). 2021;100:e24005. 10.1097/MD.0000000000024005 PMC7870182 33592856

[6] Xu G Zhu G Jiang X Cui J Liang Z. Endoscopic revision for long-term symptomatic cage retropulsion after TLIF: the clinical presentation in a single center. Orthop Surg. 2023;15:1210–5. 36788444 10.1111/os.13668 PMC10102315

[7] Tanaka M Wei Z Kanamaru A. Revision for cage migration after transforaminal/posterior lumbar interbody fusion: how to perform revision surgery? BMC Surg. 2022;22:172. 35546229 10.1186/s12893-022-01620-0 PMC9092779

[8] Al-Rabiah AM Alghafli ZI Almazrua I. Using an extreme lateral interbody fusion (XLIF) in revising failed transforaminal lumbar interbody fusion (TLIF) With exchange of cage. Cureus. 2021;13:e14123. 33927931 10.7759/cureus.14123 PMC8075769

[9] Ng BW Baharuddin A Tan JA Muhamad Ariffin MH. Revision spinal surgery for posterior migration of tantalum cage: tips and tricks. Cureus. 2022;14:e23794. 35530865 10.7759/cureus.23794 PMC9067354

[10] Qin R Wu T Liu H Zhou B Zhou P Zhang X. Minimally invasive versus traditional open transforaminal lumbar interbody fusion for the treatment of low-grade degenerative spondylolisthesis: a retrospective study. Sci Rep. 2020;10:21851. 33318543 10.1038/s41598-020-78984-x PMC7736320

[11] Kim PD Baron EM Levesque M. Extrusion of expandable stacked interbody device for lumbar fusion: case report of a complication. Spine. 2012;37:E1155–8. 22498990 10.1097/BRS.0b013e318257f14d

[12] Kim M-C Chung H-T Cho J-L Kim D-J Chung N-S. Subsidence of polyetheretherketone cage after minimally invasive transforaminal lumbar interbody fusion. J Spinal Disord Tech. 2013;26:87–92. 23529151 10.1097/BSD.0b013e318237b9b1

[13] Yun D-J Yu J-W Jeon S-H Lee H-C Lee S-H. Salvage anterior lumbar interbody fusion for pseudoarthrosis after posterior or transforaminal lumbar interbody fusion: a review of 10 patients. World Neurosurg. 2018;111:e746–55. 29309972 10.1016/j.wneu.2017.12.155

[14] Xie B Zhu G Shen G. Endoscopic resection and decompression for lateral displacement of cage after oblique lumbar interbody fusion: a case report from a single center. Orthop Surg. 2023;15:2730–5. 37435856 10.1111/os.13808 PMC10549870

[15] Ekşi MS Topçu A Topaloğlu F. Fatty infiltration in the multifidus predicts screw-loosening following short-segment decompression and fusion: proof of why we should protect and rehabilitate the paraspinal muscles. Eur Spine J. 2025;34:2427–37. 40140014 10.1007/s00586-025-08793-1

